# Telemedicine and Virtual Reality for Cognitive Rehabilitation: A Roadmap for the COVID-19 Pandemic

**DOI:** 10.3389/fneur.2020.00926

**Published:** 2020-09-15

**Authors:** Elisa Mantovani, Chiara Zucchella, Sara Bottiroli, Angela Federico, Rosalba Giugno, Giorgio Sandrini, Cristiano Chiamulera, Stefano Tamburin

**Affiliations:** ^1^Department of Neurosciences, Biomedicine and Movement Sciences, University of Verona, Verona, Italy; ^2^Section of Neurology, Department of Neurosciences, Verona University Hospital, Verona, Italy; ^3^Giustino Fortunato University, Benevento, Italy; ^4^IRCCS Mondino Foundation, Pavia, Italy; ^5^Department of Computer Science, University of Verona, Verona, Italy; ^6^Department of Brain and Behavioral Sciences, University of Pavia, Pavia, Italy; ^7^Department of Diagnostics and Public Health, University of Verona, Verona, Italy

**Keywords:** augmented reality, cognitive, COVID-19, rehabilitation, telemedicine, virtual reality

## Abstract

The current COVID-19 pandemic presents unprecedented new challenges to public health and medical care delivery. To control viral transmission, social distancing measures have been implemented all over the world, interrupting the access to routine medical care for many individuals with neurological diseases. Cognitive disorders are common in many neurological conditions, e.g., stroke, traumatic brain injury, Alzheimer's disease, and other types of dementia, Parkinson's disease and parkinsonian syndromes, and multiple sclerosis, and should be addressed by cognitive rehabilitation interventions. To be effective, cognitive rehabilitation programs must be intensive and prolonged over time; however, the current virus containment measures are hampering their implementation. Moreover, the reduced access to cognitive rehabilitation might worsen the relationship between the patient and the healthcare professional. Urgent measures to address issues connected to COVID-19 pandemic are, therefore, needed. Remote communication technologies are increasingly regarded as potential effective options to support health care interventions, including neurorehabilitation and cognitive rehabilitation. Among them, telemedicine, virtual reality, augmented reality, and serious games could be in the forefront of these efforts. We will briefly review current evidence-based recommendations on the efficacy of cognitive rehabilitation and offer a perspective on the role of tele- and virtual rehabilitation to achieve adequate cognitive stimulation in the era of social distancing related to COVID-19 pandemic. In particular, we will discuss issues related to their diffusion and propose a roadmap to address them. Methodological and technological improvements might lead to a paradigm shift to promote the delivery of cognitive rehabilitation to people with reduced mobility and in remote regions.

## Introduction

Disorders of cognitive functions (language, perception, attention, memory, executive functions, and praxis) are frequent following neurological damage of different etiology, with a significant impact on independence, social relationships, school attendance, and employment opportunities, ultimately leading to reduced quality of life. Cognitive impairment is a critical determinant of overall neurorehabilitation outcome, and cognitive rehabilitation is an expanding clinical and research field.

Cognitive rehabilitation encompasses a wide range of therapeutic cognitive interventions to achieve functional changes by reinforcing, strengthening, or reestablishing previously learned patterns of behavior or establishing new patterns of cognitive activity or mechanisms to compensate for impaired neurological systems (1). These interventions are based on psychological theories and models of behavior and behavioral change and on neuropsychological models of brain–behavior interactions (2, 3), and can be conducted with paper–pencil tools, computer programs, or, more recently, virtual reality (VR).

Several works explored the effectiveness of cognitive rehabilitation. While some studies adopted a pragmatic clinical focus, supporting the efficacy of neuropsychological interventions (4, 5), other reports emphasized the lack of methodological rigor of trial design, concluding that there is insufficient evidence to guide the clinical practice (6–10). To overcome these limitations, the Cognitive Rehabilitation Task Force (CRTF) of the American Congress of Rehabilitation Medicine, Brain Injury Special Interest Group, recently published a systematic review of studies addressing cognitive rehabilitation for people with two of the most frequent clinical conditions, namely, stroke and traumatic brain injury (TBI) (11). The authors evaluated 491 articles and made 29 recommendations for evidence-based practice of cognitive rehabilitation that support practice standards for (1) attention deficits after TBI or stroke; (2) visual scanning for neglect after right-hemisphere stroke; (3) compensatory strategies for mild memory deficits; (4) language deficits after left-hemisphere stroke; (5) social communication deficits after TBI; (6) metacognitive strategy training for deficits in executive functioning; and (7) comprehensive–holistic neuropsychological rehabilitation to reduce cognitive and functional disability after TBI or stroke (11).

To be effective, cognitive rehabilitation should be intensive and prolonged over time, but social events that reduce access to care facilities hamper intensive and prolonged cognitive rehabilitation, unless current protocols are modified. This is the case we have been dealing with since December 2019, when a pneumonia epidemic of previously unknown etiology in China was related to SARS-CoV-2 infection. In March 2020, the World Health Organization declared the COVID-19 pandemic (12). Since then, the virus has spread widely and rapidly. On June 4, 2020, more than 6 million cases of COVID-19, and nearly 380 hundred deaths have been reported worldwide (13). In the absence of an effective treatment against SARS-CoV-2, the outbreak containment strategies mainly rely on hygienic measures, extraordinary sanitization, and reduction of interpersonal contacts through social distancing and quarantine for infected people and their contacts (14). In this scenario, healthcare systems need to reorganize quickly and deeply both in the wards hosting COVID-19 patients and in the services for patients with chronic diseases. Social distancing and quarantine, indeed, abruptly interrupted access to routine medical care for frail and vulnerable people, who are at an increased risk of SARS-CoV-2 infection and related morbidity and death. Patients with neurological diseases are among such frail patients because of advanced age, comorbidities, or immunosuppression due to treatments (15). In addition, the best medical practices have also been suspended for patients whose doctors have been in quarantine or for people with stroke and myocardial infarction, who have not sought medical treatment for fear of social contact (16, 17).

Therefore, timely measures are required to mitigate the potentially harmful consequences of quarantine, and telemedicine approach to achieve non-face-to-face consultations has been proposed (18).

We will review features of telerehabilitation, VR, and other technologies to achieve cognitive telerehabilitation (Table 1); provide some suggestions to enhance cognitive rehabilitation interventions during the COVID-19 pandemic; and propose future implementations based on telemedicine and VR.

**Table 1:** Main methods and technologies for cognitive telerehabilitation.

	**Definition**	**Advantages**	**Limitations**
Telerehabilitation	The provision of rehabilitation services via telemedicine methods and techniques (19)	Increases frequency of healthcare professional contact Facilitates intensive and prolonged programs Allows the access to home-delivered care	Barriers to accessing technologies (e.g., lack of computer or internet connection) in specific patient groups (e.g., elderly people)
Virtual Reality	A computer-based, interactive, multisensory environment that occurs in real time, with which the user can directly interact (20)	Provides immediate feedback Allows the adaptation to patient's performance Highly engaging High level of ecological validity Can be combined with other tools/devices (e.g., electroencephalography, physiological activity registration tools)	Technology requirements are often cumbersome Limited availability (i.e., outpatient clinics) Expensive hardware and software tools
Augmented Reality	The overlaying of computer-generated imagery atop the real world using a see-through display (21)	Employs wearable devices Allows the adaptation to patient's performance High patient engagement Available for home-delivered care	Limited user's immersion Barriers to accessing technology
Serious Games	Digital games whose purpose is to reach a specific goal (e.g., cognitive rehabilitation) other than entertainment (22)	Allows the adaptation to patient's performance High patient engagement Affordable costs Available for home-delivered care	Lack of immersion Limited flexibility and customizing

## Telemedicine and Telerehabilitation for Cognitive Disorders

Telemedicine is a general term, first introduced in the 1970s, to indicate the practice of medicine without the usual physical interaction between a healthcare professional and a patient using an interactive multimedia communication system (23). Telemedicine includes the application of information and communication technology (ICT) to the medical field to guarantee remote assistance services based on the exchange of clinical information and data within a network of professionals or between professionals and clients (24). In parallel to the classical doctor–patient relationship, telemedicine must comply with all the rights and duties of any health act for prevention, diagnosis, treatment, rehabilitation, and monitoring. Telemedicine is not meant to replace traditional health services but rather to integrate them to improve effectiveness, efficiency, and appropriateness (25).

Stemming from the broader approach of telemedicine, telerehabilitation is an alternative method of delivering conventional rehabilitation services via ICT to patients allowing them access to care at their homes or other locations (19, 26). Telerehabilitation systems provide therapists with the possibility of selecting the most appropriate approach for each individual patient, monitoring execution and outcomes remotely, and modifying the treatment accordingly. The COVID-19 pandemic has accelerated this process and forced researchers and clinicians to reshape the neurorehabilitation strategies with the use of technologies (27) and to accelerate the development of telemedicine for home care purposes, e.g., the use of low-cost technologies such as smartphones or tablets for virtual medical examination, counseling, and rehabilitation (15, 28).

Tele-health approaches were demonstrated to be feasible, well-accepted, and effective in providing rehabilitation to chronic neurological patients, increasing participation, and allowing the continuity of care in an ecologic environment (29).

Telerehabilitation was initially aimed to improve motor outcomes, but the interest in the treatment of cognitive deficits has increased over the years. Studies ranged from pilot reports, assessing the feasibility of postoperative telerehabilitation programs to improve cognitive outcomes in adult patients with primary brain tumors (30), to systematic reviews and meta-analyses focused on neurodegenerative disorders (31, 32), stroke (33), and multiple sclerosis (34).

Telemedicine interventions were found not to be inferior to conventional face-to-face approaches in terms of efficacy, validity, reliability, and patients' satisfaction, but the low number of randomized controlled trials hampered definitive conclusions (35, 36). Based on these promising results and forced by COVID-19 contingency, new studies and a larger diffusion of cognitive telerehabilitation approaches are expected.

## Virtual Reality for Cognitive Rehabilitation

Over recent years, researchers and clinicians proposed VR as a new technology to implement innovative treatments in a broad range of clinical areas, including mental health disorders (e.g., anxiety disorders, depression, schizophrenia, eating disorders), pain management (37–41), motor and cognitive rehabilitation of neurodegenerative disorders, TBI and stroke (42), and cognitive domains (43–46).

VR allows the user to interact with, and become immersed in, a computer-generated environment in a naturalistic way. The key concepts that define VR are immersion (i.e., the extent to which the user perceives himself in the virtual environment rather than the real world), sense of presence (i.e., the subjective experience of the user as being in the virtual world), and the possibility to interact with the computer-generated environment (20, 47, 48).

VR has a number of advantages over traditional rehabilitation approaches. First, VR has a high level of ecological validity because of the sensorimotor interaction between the user and the virtual environment, allowing to transfer skills from virtual to real word. Second, the compliance and the satisfaction of the patient when interacting with the enriched computer-generated environment are higher than those with conventional rehabilitation (49). Third, VR has the great advantage of providing an immediate and direct feedback, so that the level of difficulty of the therapy can be easily adapted to the patient's needs and severity (50), with positive effects on their sense of efficacy. By providing quantitative outcome measures to patients, VR supports better adherence to neurorehabilitation programs than to traditional rehabilitation (51). Fourth, VR rehabilitation programs can be applied without the direct supervision of the therapist, but only with the presence of a caregiver (44), addressing the patient's need of autonomy. Fifth, VR allows patients to perform basic daily living activities in a safe and controlled environment, increasing engagement and motivation (52). This is particularly important, considering that traditional training programs are often repetitive and monotonous. VR may engage the patient in an enriched environment and stimulating activities, thus activating attention and motivation, and facilitating neuroplasticity and functional recovery (53, 54). VR research protocols are increasingly applied to rehabilitation, as technology becomes more accessible and affordable, but VR is not yet routinely used in clinical rehabilitation settings because of several issues. The term VR is frequently used in the wrong way, as some studies improperly define computer-based devices providing stimuli on a monitor (e.g., video games), which clearly lack two out of the three key features of VR, i.e., immersion and presence. As gaming consoles are widely available, clinicians have indeed started to use low-cost commercial immersive systems designed for recreation as an alternative way of delivering VR (55–57), but the lack of specific VR features may result in a limited therapeutic effect of these devices. Moreover, VR systems are often cumbersome and expensive, thus hampering the possibility to perform VR rehabilitation interventions outside the outpatient clinics (44). The possibility to perform immersive VR-based rehabilitation programs at home is an important challenge that should be addressed in the near future. Moving from a single-user VR setting available in the clinic to a multiuser one with remote connection between patients/caregivers and therapists could be an important step toward the dissemination of VR technologies (58).

Among graphic immersive techniques, augmented reality (AR) is another novel technological system that enhances the sensory experience of the real environment by inserting virtual elements to the view of the physical environment, usually using a camera, smartphone, or other vision devices (21). In contrast to VR, AR environment is not completely computer generated but is a combination of real and virtual objects in a physical environment (59). The amplification of sensory experience through AR was found to be associated with a significant improvement of the ecological validity of treatments of various health disorders (60). AR-based treatment has been proposed for phobic disorders and stroke (61, 62).

More recent approaches include serious games (SGs), i.e., interactive computer applications, in which education and learning, not entertainment, are the primary goals (63, 64). Due to their design, games can offer challenging, rewarding, motivating, and engaging experiences that can be shared with other players in the form of points or ranking. Indeed, the interactive nature of the games enables constructive, situational, and experiential learning opportunities that can be easily adopted for rehabilitation purposes, despite not having been fully designed for rehabilitation goals (65). SG-based treatments derive from the combination of specific elements of computer cognitive training with motivational aspects of games (66). Similar to AR, SGs are characterized by an immersive level of each environment that can range from the complete VR to the real environment (67). Hence, the smaller computation time required to model the 3D environment of AR and SG may make them more cost effective in comparison to VR (61). Most SG-based cognitive treatments have been directed to healthy older adults or patients with mild cognitive impairment or Alzheimer's dementia (68). The fact that elderly people could have difficulties in interacting with tools designed for the game (69) has determined a recent interest to develop SGs specifically designed for these populations (66). Since cognitive rehabilitation adopts a restitution-based approach, in which impaired functions, either physical or cognitive, are recovered through intense and continuous stimulation (70), SG-based interventions are particularly useful to this end, being available also for home-based rehabilitation (71). Cognitive treatments using SGs have been developed also for stroke, TBI, brain tumors (72) and cerebral palsy (73). In conclusion, even if SG systems are appealing because of their low cost, their diffusion is partially limited because of the lack of customization and of rehabilitation theoretical models behind their development. The smaller computation time required to model the 3D environment of AR and SG may make them more cost effective in comparison to VR (61), overcoming some VR limitations and providing another option for remote cognitive rehabilitation.

## Discussion

**Figure 1:**
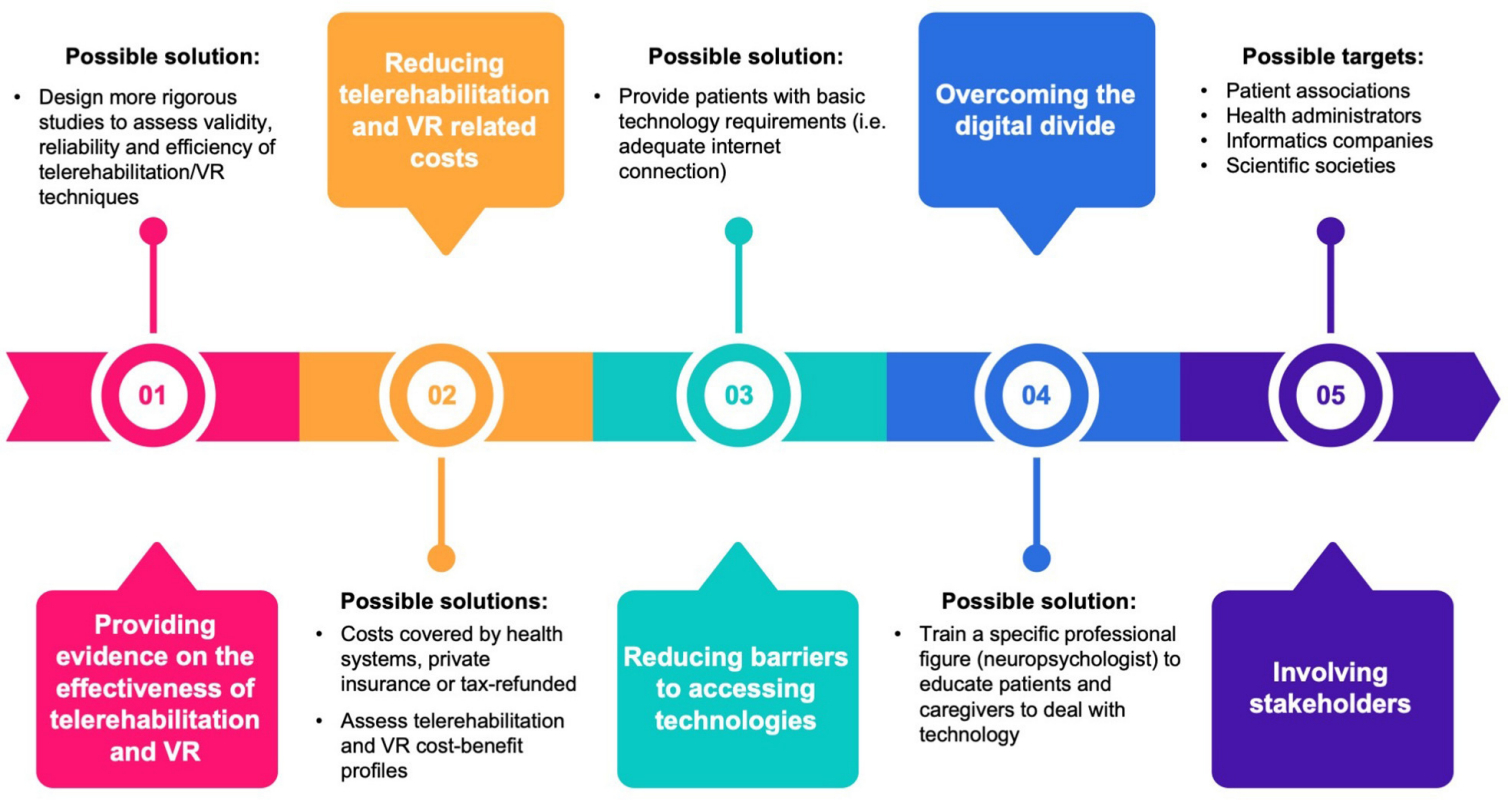
Issues related to cognitive telerehabilitation and possible solutions. VR, virtual reality.

The current health system contingency due to the COVID-19 pandemic requires an acceleration in the use of telemedicine to enable cognitive neurorehabilitation outside the traditional settings (e.g., hospital, rehabilitation centers, private practice) and in an ecologic environment. Teletherapy may replace and complement in-person treatment to mitigate constraints on service delivery that currently limit access to cognitive rehabilitation care. Telemedicine, VR, AR, and SGs are promising tools for remote-delivered cognitive rehabilitation programs. There are, however, a number of open questions that hamper these approaches to become a valid complement to standard care of patients with cognitive deficits. We propose a roadmap to address these issues (Figure 1).

First, evidence supporting telerehabilitation and VR for cognitive rehabilitation is still preliminary, and a larger number of studies focusing on the validity, reliability, effectiveness, and efficiency of these techniques and approaches are needed. The use of VR therapy is indeed far from becoming widespread beyond the research setting, thus limiting its translation into the ordinary clinical setting (74). Another point that limits the spread of telerehabilitation and VR for cognitive rehabilitation beyond the research setting is that these techniques have no specific effect on a single (e.g., executive, visuospatial, and memory) domain, but they are rather intended to stimulate at the same time multiple domains to achieve high levels of ecological validity. The development of more targeted and specific VR and telerehabilitation techniques to be compared with “traditional” ones could offer challenging opportunities for future research. Moreover, the lack of specific clinical training in VR therapy could be another issue that hampers its diffusion (75). The identification of specific health professional figures (e.g., neuropsychologists) to be adequately trained could be a possible solution. An important point to be investigated to contribute to the dissemination of VR therapy is the tolerance of VR interventions, i.e., the gradual decrease in effect due to the lack of novelty of the experience. A critical component is safety and tolerability: VR sickness and boredom should be monitored to avoid dropouts and lack of compliance.

Second, the high cost of the hardware and software required for these techniques is still a bottleneck that impedes their wide application outside the experimental setting. Moreover, these costs are covered neither by health systems and private insurance nor by tax refund. Studies exploring their cost–benefit profiles in terms of reduced direct and indirect costs related to cognitive deficits might help overcome this issue. A wider diffusion of hardware platforms and the use of open software might consistently reduce these costs, in analogy to what happened in recent years with mobile phones and consumer technology.

Third, a high-speed Internet connection is of paramount importance to improve telerehabilitation and remote monitoring from the therapist, but in some areas, it may not be available.

Fourth, the digital divide in some countries/regions, in older adults, and in some classes of people might reduce the wide application of cognitive telerehabilitation. A specific figure, i.e., the neuropsychologist with expertise in these techniques, including the ability to remotely monitor the correct application of cognitive telerehabilitation at home, educate caregivers, and help them to solve technical issues, would be important to reduce the effects of this digital divide.

Addressing these points requires the involvement of a number of stakeholders, including patient associations, health, informatics, and scientific societies, but may result in a consistent improvement in cognitive rehabilitations strategies in that carrying out interventions at home is even more important because the generalization of the results to daily life activities is one of the most critical elements for the success of the intervention. Addressing the abovementioned issues may lead to a wider application of teletherapy, e.g., to the still unexplored area of behavioral and psychological symptoms of dementia (BPSD) management. Because of the limited benefits of the pharmacological and nonpharmacological interventions (e.g., environmental redesign, validation therapy, and behavioral management techniques) for BPSD (64), telemedicine, and VR may offer new options for this condition. Preliminary results are, indeed, encouraging, either for patients (76, 77) or caregivers (78–80).

Methodological and technological improvements might survive the end of the COVID-19 pandemic and result in a cost-effective and sustainable paradigm shift for remote delivering of health services to people with reduced mobility and access to hospitals and rehabilitation centers, and in remote regions not covered by these facilities. Adapting healthcare facilities during the COVID-19 pandemic through new technology could help support the cognitive and psychosocial needs of both patients and their families (81).

## Data Availability Statement

The data analyzed in this study is subject to the following licenses/restrictions: The datasets generated for this study are available on request to the corresponding author. Requests to access these datasets should be directed to ST, stefano.tamburin@univr.it.

## Author Contributions

The study has been designed by EM, CZ, CC, and ST. Data have been gathered and analyzed by EM, CZ, SB, AF, RG, GS, CC, and ST. The manuscript has been drafted by EM, CZ, SB, AF, CC, and ST. SB, RG, GS, CC, and ST revised the manuscript. All Authors approved the final version of the manuscript.

## Conflict of Interest

The authors declare that the research was conducted in the absence of any commercial or financial relationships that could be construed as a potential conflict of interest.
